# Skeletal Muscle and the Maintenance of Vitamin D Status

**DOI:** 10.3390/nu12113270

**Published:** 2020-10-26

**Authors:** Mark S. Rybchyn, Myriam Abboud, David A. Puglisi, Clare Gordon-Thomson, Tara C. Brennan-Speranza, Rebecca S. Mason, David R. Fraser

**Affiliations:** 1 Department of Physiology, School of Medical Sciences and Bosch Institute, Faculty of Medicine and Health, The University of Sydney, Sydney, NSW 2006, Australia; mark.rybchyn@sydney.edu.au (M.S.R.); Myriam.abboud@zu.ac.ae (M.A.); dpug9928@alumni.sydney.edu.au (D.A.P.); clare.gordon-thomson@sydney.edu.au (C.G.-T.); tara.speranza@sydney.edu.au (T.C.B.-S.); rebecca.mason@sydney.edu.au (R.S.M.); 2 Department of Health Sciences, College of Natural and Health Sciences, Zayed University, Dubai, Abu Dhabi P.O. Box 144534, UAE; 3 School of Public Health, Faculty of Medicine and Health, The University of Sydney, Sydney, NSW 2006, Australia; 4 Sydney School of Veterinary Science, Faculty of Science, The University of Sydney, Sydney, NSW 2006, Australia

**Keywords:** vitamin D, muscle, parathyroid hormone, vitamin D-binding protein

## Abstract

Vitamin D, unlike the micronutrients, vitamins A, E, and K, is largely obtained not from food, but by the action of solar ultraviolet (UV) light on its precursor, 7-dehydrocholesterol, in skin. With the decline in UV light intensity in winter, most skin production of vitamin D occurs in summer. Since no defined storage organ or tissue has been found for vitamin D, it has been assumed that an adequate vitamin D status in winter can only be maintained by oral supplementation. Skeletal muscle cells have now been shown to incorporate the vitamin D-binding protein (DBP) from blood into the cell cytoplasm where it binds to cytoplasmic actin. This intracellular DBP provides an array of specific binding sites for 25-hydroxyvitamin D (25(OH)D), which diffuses into the cell from the extracellular fluid. When intracellular DBP undergoes proteolytic breakdown, the bound 25(OH)D is then released and diffuses back into the blood. This uptake and release of 25(OH)D by muscle accounts for the very long half-life of this metabolite in the circulation. Since 25(OH)D concentration in the blood declines in winter, its cycling in and out of muscle cells appears to be upregulated. Parathyroid hormone is the most likely factor enhancing the repeated cycling of 25(OH)D between skeletal muscle and blood. This mechanism appears to have evolved to maintain an adequate vitamin D status in winter.

## 1. Characterization of Vitamin D Status

The concentration in blood serum or plasma of 25-hydroxyvitamin D [25(OH)D], which is the most plentiful vitamin D metabolite, has become established as the definitive indicator of vitamin D status [1,2]. When the concentration falls below a generally agreed level, (usually 50 nmol/L), vitamin D status is said to be insufficient or deficient in the same way that status is defined for other small molecules derived from the environment, such as vitamins A, E, and K. However, there are three substantial differences between this measurement of vitamin D status and measurements of these fat-soluble micronutrients. The first is that 25(OH)D concentration in blood varies with season. Since it is derived from vitamin D produced in skin by the action of solar ultraviolet radiation on 7-dehydrocholesterol, the 25(OH)D levels rise during the months of summer and fall during winter, particularly in those people who live far from the equator [3,4]. In temperate geographical regions in winter, the intensity of solar UV light is very low in the vitamin D-producing wavelength range of 290–320 nm [5,6]. Are the lower values of 25(OH)D concentration in winter really an indication of deficiency or insufficiency of vitamin D, if this is a universal feature of populations?

The second difference with vitamins A, E, and K is that there is no apparent storage organ or tissue for vitamin D or 25(OH)D. Although vitamin D is found in adipose tissue, suggesting that this is a storage site [7,8], it can only be released when stored fatty acids are mobilized to supply energy [9,10,11]. Thus, sequestered vitamin D in adipocytes should not be regarded as a functional store, ready to be transported to the liver and converted to 25(OH)D, whenever circulating levels of this metabolite decline.

The third unique feature is that 25(OH)D has a very long residence time in blood. The half-life is very variable between 15 and 50 days [12] with a mean value in a recent study of 89 days [13]. In contrast, other hormonal steroids in blood, including the vitamin D hormone, 1,25-dihydroxyvitamin D (1,25(OH)_2_D), are cleared in minutes or hours after entering the circulation [14,15]. An explanation for this long residence time of 25(OH)D is not readily apparent, particularly because the vitamin D binding protein (DBP) in blood, to which 25(OH)D is tightly bound, has a comparatively short half-life of only 1–3 days and is continuously being replenished from synthesis and secretion by the liver [16,17,18]. Therefore, for 25(OH)D to be retained in blood for such a long time, it either has to transfer from one DBP molecule to another in the circulation or else it repeatedly passes to and from some extravascular site, binding to a new DBP molecule with each cycle. The DBP concentration in blood is in vast excess to that of 25(OH)D with only 1%–5% of the protein molecules having a 25(OH)D molecule bound to the single, high affinity, specific binding site [19].

This article reviews new evidence for a conservation mechanism for 25-hydroxyvitamin D by skeletal muscle cells.

## 2. Evidence for Conservation of 25(OH)D

The discovery that maternal 25(OH)D in rats was transported across the placenta and accumulated in the skeletal muscle of fetuses suggested that skeletal muscle might have a functional role in conserving this vitamin D metabolite [20]. Although the concentration of 25(OH)D in the muscle of sheep and cattle is only about 0.1–0.3 µg/100 g [21], muscle represents 30%–38% of body mass in humans [22] and, thus, total 25(OH)D in total skeletal muscle could be comparable to that in the circulation (Table 1).

In addition, studies in adolescent children [24] found that plasma 25(OH)D concentration was positively correlated with total body lean mass in which the main component is skeletal muscle. Furthermore, there are now several published findings of a positive relationship between the intensity of physical exercise and the concentration of 25(OH)D in blood, e.g., [25,26], particularly in winter when there would be little exposure to solar UV light during outdoor exercise [24]. Muscle biopsies from sheep grazing outdoors in winter showed a significantly higher concentration of 25(OH)D than biopsies from sheep outdoors at the end of summer (Table 2).

## 3. Skeletal Muscle Cell Uptake of DBP

The key discovery that pointed to a role for muscle in maintaining vitamin D status came from studying the properties of muscle cells in vitro [28]. The cell membrane was found to contain the proteins megalin and cubilin. These proteins, like those in the renal tubule cells [29,30] and in hepatic stellate cells [31], transport DBP from the extracellular fluid into the cell cytoplasm. The vitamin D binding protein has two specific, high-affinity binding sites. One is for vitamin D and its metabolites with the highest affinity being for 25(OH)D (Kd < 1 nmol/L). The other binding site is specific for filamentous actin [32,33]. A commonly held theory postulates that the actin-binding site of DBP functions to bind actin if released into blood from damaged cells and, thus, protects against intravascular coagulation [34,35]. Yet it has been known for over 30 years that DBP becomes bound to actin in skeletal muscle [36]. Some of the internalized DBP could be bound to actin in actomyosin, via its specific actin-binding site, but much of the remainder is bound to actin dispersed throughout the cytoplasm. Both megalin and DBP have also been found in human muscle biopsies [37].

The role attributed to megalin/cubilin in renal proximal tubule cells is the recovery of DBP that leaks into the glomerular filtrate. This process is credited with conserving 25(OH)D even though only 1%–5% of the retrieved DBP molecules would be transporting 25(OH)D [19]. The observation that genetically modified global megalin-knockout mice become vitamin D deficient has, therefore, been interpreted as evidence that renal megalin uptake of DBP prevents loss of DBP-bound 25(OH)D in urine [29]. A more plausible explanation is that muscle conservation of 25(OH)D is completely abolished in megalin-knockout mice so that the residence time of 25(OH)D in blood would be no greater than that of DBP itself. The main site of DBP clearance from blood has also been attributed to the kidney [33]. Yet, the uptake and proteolysis of DBP in the much larger mass of total body skeletal muscle would appear to be a more likely explanation for its short residence time in the circulation.

**Figure 1:**
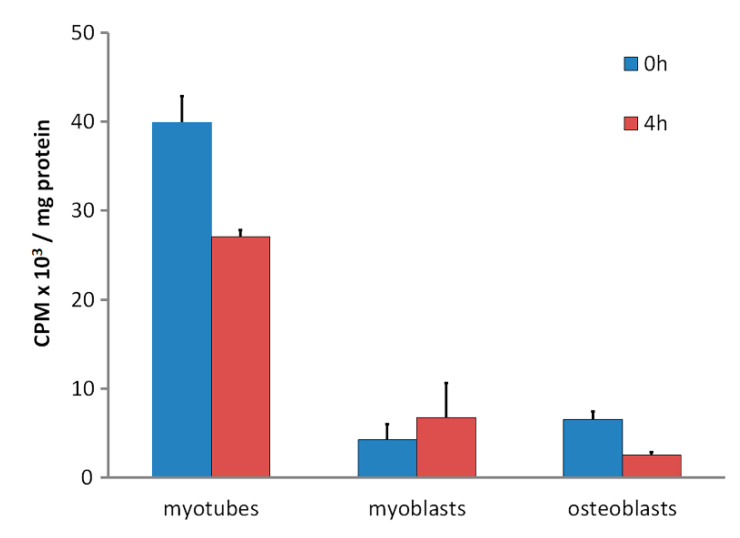
(Data reformatted from Reference [28]). Retention of tritiated-25(OH)D3 in C2 myotubes, C2 myoblasts and MG63 osteoblasts. Cells were incubated for 16 h with [26,27-^3^H]25-hydroxyvitamin D3 (Perkin Elmer, Waltham, MA, USA) in Dulbecco’s Modified Eagle’s Medium (DMEM) supplemented with serum replacement (Sigma-Aldrich, St. Louis, MO, USA) followed by 3x washes with ice cold Phosphate Buffered Saline (PBS). At this point, 0 h or 4 h later, cells were harvested, lysed, and assayed for protein by the bicinchoninic acid assay (Thermo-Scientific, Bannockburn, IL, USA) or radioactivity counted by liquid scintillation counting (see Reference [28]). Data as mean counts per minute (CPM) ± Standard Error of the Mean (SEM) (*n* = 3 wells/group).

In vivo experiments with ^131^I-labelled DBP in rabbits revealed that DBP had a short residence time in skeletal muscle and soon underwent proteolysis [16]. DBP is a member of the albuminoid family of proteins. It is synthesized and secreted by the liver with similar characteristics to blood albumin [33]. Because albumin in the extracellular fluid in skeletal muscle is only about one-third the concentration in plasma [38], the extracellular DBP concentration will likewise be decreased to about 1.5 to 2 µmol/L when compared to the blood concentration of 5–6 µmol/L [39]. Thus, much of the 25(OH)D in the extracellular fluid will have dissociated from the low concentration of DBP and would be able to diffuse into muscle cells where it would bind to the internalized DBP in the cell cytoplasm. In comparison with control cells such as osteoblasts, 25(OH)D, is retained within myotubes (the differentiated cell type derived from myoblasts in culture, equivalent to myocytes in vivo) for a considerably longer time (Figure 1). When cytoplasmic DBP undergoes proteolysis in vivo, the bound 25(OH)D is released and would diffuse from the myocyte and return to the circulation, once again being bound to the plentiful DBP. This repeated uptake and release of 25(OH)D by the total mass of skeletal muscle cells would account for the apparent long residence time of 25(OH)D in blood plasma.

An alternative explanation for the long half-life of 25(OH)D in blood could be that there is continuous entry of newly synthesized 25(OH)D, perhaps from parent vitamin D trapped in adipose tissue. This possible steady input could be replacing a steady loss of 25(OH)D from blood, and, thus, account for an apparently long half-life. However, the long residence time in blood of tritium-labelled 25(OH)D in both mice [28] and humans [12] demonstrates that it is persistence of the same molecules in blood that is the real explanation. This phenomenon can only be explained by recycling of 25(OH)D from some extravascular region and skeletal muscle is the only candidate for that region.

Nevertheless, experiments investigating the uptake and retention of 25(OH)D in cultured muscle cells in vitro, have shown that raising the concentration of DBP in the culture medium blocks the uptake of 25(OH)D into the cells (Table 3). The higher the concentration of DBP, the lower the concentration of unbound 25(OH)D. This observation fits with the interpretation that it is unbound 25(OH)D that enters muscle cells, rather than that which might be carried in, bound to DBP, via the megalin/cubilin protein internalization process. The fact that only 1%–5% of DBP in the circulation is transporting a specifically bound 25(OH)D molecule also indicates that transport on DBP across the cell membrane cannot be the mechanism for the intracellular accumulation of 25(OH)D.

**Figure 2:**
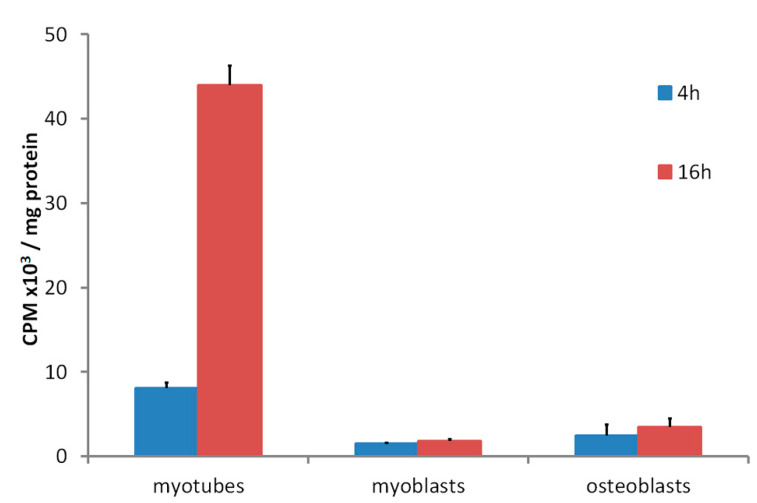
(Data reformatted from Reference [28]) Time dependent tritiated-25(OH)D uptake in C2 myotubes and myoblasts and MG63 osteoblasts. Cells were incubated for 4 or 16 h with [26,27-^3^H]25-hydroxyvitamin D_3_ in DMEM supplemented with serum replacement and followed by 3x washes with ice cold PBS. At each time point, cells were harvested, lysed, and assayed for protein by the bicinchoninic acid assay or radioactivity counted by liquid scintillation counting (see Reference [28]). Data shown as means ± SEM (*n* = 3 wells/group).

**Figure 3:**
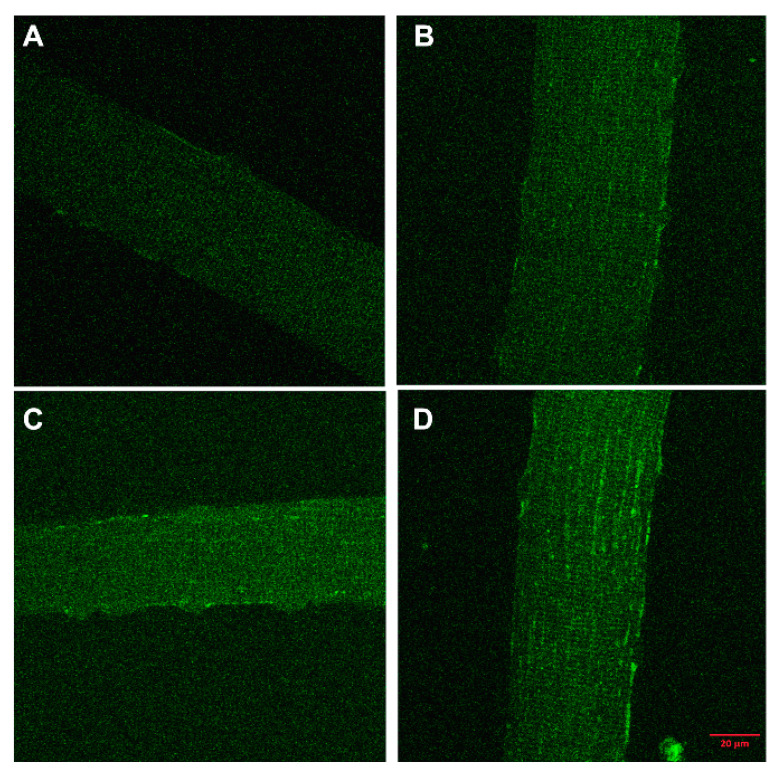
(Images referred to in Reference [28]). The uptake of Alexafluor488-labelled D-binding protein (DBP) into primary muscle fibers. Myofibers were isolated from the flexor digitorum brevis of euthanized Balb/c mice and incubated in quadruplicate wells on poly-L-lysine-coated glass coverslips. Fibers were incubated for 0, 4, 12, or 24 h with 1 µmol/L of Alexa Fluor488-DBP (labelling kit from Molecular Probes) in DMEM supplemented with 20% DBP-free serum replacement. Incubations were set up to finish at the same time. Cells were then fixed in paraformaldehyde for 20 min. Fluorescent images were captured with a Zeiss LSM 510 Meta Spectral confocal microscope, using optimized saturation settings for the 24-h time point. The fluorescence intensity can be seen increasing from (**A**) 0 h, (**B**) 4 h, (**C**) 12 h, and (**D**) 24 h.

The accumulation of tritium-labelled 25(OH)D over 16 h by cultures of differentiated mouse skeletal myotube cells compared to the very low uptake by undifferentiated myoblasts and osteoblasts [40] demonstrated that some internal 25(OH)D-specific binding sites were present in the myotubes but not in the other two control cell types (Figure 2). Since the culture medium during the time of these incubations did not contain DBP, the specific binding inside myotubes would have been to DBP, which had been internalized from the culture medium while the myotubes were differentiating from myoblasts. When myotubes were cultured with fluorescently labelled DBP for up to 24 h and then observed by confocal microscopy, the fluorescent protein was clearly seen within the cells (Figure 3). Some of the fluorescence was in linear streaks along the long axis of the myotubes, suggesting that some of the labelled DBP was bound to actin in the actomyosin contractile elements. However, the fluorescent pattern was also distributed generally within the cell, indicating that much of the DBP was bound to the abundant actin, known to be distributed throughout the myotube cytoplasm [41].

It now appears that the megalin/cubilin protein internalization mechanism in myocytes is not limited to DBP because other extracellular proteins, such as albumin, have been found in human skeletal muscle [37] and have also been demonstrated to be incorporated into myotube cells in culture by this mechanism (unpublished results). Thus, the endocytosis of albumin by megalin/cubilin in muscle cells is similar to this property of megalin/cubilin in renal proximal tubule cells [30]. The function of the uptake of other extracellular proteins by skeletal muscle cells in vivo is a matter of speculation. Since skeletal muscle has a high rate of protein turnover, particularly when undergoing regular physical exercise [42], the internalized proteins, including DBP, could, after proteolysis, be supplying essential amino acids for protein resynthesis. It is, therefore, conceivable that, because DBP in myotube cultures is capable of retaining 25(OH)D for many hours (Figure 1) [28], the DBP bound to cytoplasmic actin is protected from the proteolysis that other internalized proteins undergo. Nevertheless, when the cytoplasmic DBP is eventually broken down, the retained 25(OH)D would be released and could then diffuse out of the cell, bind to extracellular DBP, and return to the circulation.

## 4. Regulation of Conservation Mechanism for 25(OH)D

Since the effect of regular physical exercise is associated with improved maintenance of vitamin D status in winter [24], the changes in muscle protein turnover from exercise [43] may improve the efficiency of the muscle-DBP conservation mechanism for 25(OH)D. It has been reported that a diet containing small quantities of meat gives some protection against clinical vitamin D deficiency when compared to a vegetarian diet [44]. The greater supply of essential amino acids from meat proteins compared to vegetable proteins may optimize protein turnover in muscle, which could be related to the efficiency of the conservation mechanism for 25(OH)D.

It is possible that the uptake and release of 25(OH)D by myocytes in vivo is a variable process that could be modified according to its functional role. One factor that has been shown to affect the uptake and release of 25(OH)D by myotubes in culture is the vitamin D hormone 1,25(OH)_2_D [45]. The receptor for 1,25(OH)_2_D (Vitamin D Receptor - VDR) is expressed in muscle cells, although at a low concentration [46]. Myotubes exposed to 1,25(OH)_2_D for 3 h show an enhanced accumulation of 25(OH)D over a subsequent 4-h period. However, in contrast, culturing these cells for 16 h in the presence of 1,25(OH)_2_D produces the opposite response of a decreased accumulation of 25(OH)D. Although the physiological significance of these changes is unknown, they do suggest that the uptake and release of 25(OH)D by skeletal muscle cells in vivo is potentially a VDR-regulated process.

Because the concentration of 25(OH)D in skeletal muscle of sheep (Table 2 and Reference [28]) was significantly higher in winter than in summer, during the time when plasma concentration of 25(OH)D was decreasing, it is likely that some endocrine factor was modifying the 25(OH)D retention capacity of muscle, according to changes in vitamin D status. The circulating levels of at least two hormones rise in winter and decline in summer. These are thyroid stimulating hormone (TSH) [47] and parathyroid hormone (PTH) [48]. There is no information at present to link TSH or thyroid hormone to changes in the uptake or release of 25(OH)D by skeletal muscle. However, the small increase in PTH concentration in blood plasma when 25(OH)D levels fall to 50–60 nmol/L [48], make it a candidate as a regulator of muscle uptake and release of 25(OH)D.

Receptors for PTH have now been demonstrated on mouse muscle fibers and on differentiated myotubes in culture, but receptors are not found on undifferentiated myoblast cells [27]. Incubation of myotubes with low concentrations of PTH (0.1–1 pmol/L) for 3 h, diminished the subsequent uptake of ^3^H-25(OH)D over 16 h. Conversely, the release back into the medium of ^3^H-25(OH)D, already accumulated by myotubes, was enhanced by the addition of low concentrations of PTH to the incubation medium [27]. Although these results demonstrate an effect of PTH on the transport of 25(OH)D into and out of myotubes, they paradoxically suggest that increased exposure of muscle cells to PTH would decrease their ability to conserve 25(OH)D. The rise in PTH concentration in blood as the 25(OH)D levels fall in winter suggests that, if PTH is the hormone regulating muscle uptake and release of 25(OH)D, then that enhanced uptake and delayed release of ^3^H-25(OH)D should have been seen with cells in vitro. However, in those experiments [27], the incubation medium with added PTH did not contain any DBP. Hence, the action of PTH in those studies was compatible with the hypothesis that its effect on muscle cells would be to enhance both the breakdown of cytoplasmic DBP and its rate of uptake from extracellular fluid. The effect in vivo of enhanced DBP uptake and breakdown in muscle would be to increase the rate of cycling of 25(OH)D by muscle cells and, thus, prolong the residence time of 25(OH)D in the circulation. In the disease state of hyperparathyroidism, the greatly elevated concentration of PTH in blood results in increased loss of 25(OH)D, mediated by the increased renal production of 1,25(OH)_2_D, which activates the hepatic uptake and destruction of 25(OH)D [12]. In contrast, the very small increase in PTH in blood with the seasonal decline in 25(OH)D concentration does not provoke increased secretion of 1,25(OH)_2_D from the kidney. Nevertheless, that concentration of PTH observed with the seasonal fall in vitamin D status has been shown to activate the muscle PTH receptor on myocytes in vitro [27].

## 5. Failure of Conservation Mechanism for 25(OH)D

Vitamin D deficiency, as defined by a low concentration of 25(OH)D in blood, is a common condition in populations throughout the world, and especially in those in temperate geographical regions [49]. One obvious explanation for this is underexposure of people in summer to enough solar ultraviolet-B (UVB)radiation to raise their vitamin D status to the level of sufficiency [50]. Yet, even with adequate vitamin D status at the end of summer, many people still become deficient during the following winter [51]. If the muscle mechanism for conservation of 25(OH)D has evolved to maintain vitamin D status during the annual seasonal decline in solar ultraviolet light intensity, why do so many people become vitamin D deficient?

It is notable in the limited studies on different animal species, that DBP in the blood of fish and amphibians does not have an actin-binding site [52]. Actin affinity for DBP appears to be a feature only in reptiles, birds, and mammals, which are the species that evolved as terrestrial vertebrates, dependent on solar ultraviolet light for the maintenance of vitamin D status. Furthermore, the concentration of DBP in the blood of these terrestrial animals is considerably higher than those in fish and amphibians [53,54]. This suggests that the high concentration of apo-DBP in terrestrial animals, along with its very short residence time in blood, are characteristics compatible with its function in the cycling of 25(OH)D into and out of skeletal muscle cells.

The positive association of good vitamin D status with regular physical exercise [24,25,26] suggests that the metabolic activity of muscle affects its 25(OH)D conservation ability. Another indication that changes in muscle metabolism might affect its role in conserving 25(OH)D comes from studies of the consequences of malnutrition. A high incidence of clinical vitamin D-deficiency rickets in children under 6 months of age was reported in Vienna at the end of World War I [55]. This was associated with maternal malnutrition caused by food shortages in Austria. A high frequency of rickets was similarly reported in Germany after World War II [56], again, associated with malnutrition. Rickets is also frequently seen in young children in Mongolia in winter, again, related to malnutrition caused by poverty [57].

In protein/energy malnutrition there is an increased breakdown of muscle protein to meet the needs for energy and essential amino acids [58]. If this enhanced proteolysis applies to internalized DBP in myocytes, the ability of skeletal muscle to perform its role of conserving 25(OH)D would be compromised. This could explain the high prevalence of vitamin D deficiency in malnourished people. Likewise, if the concentration of DBP in blood is depressed in humans with protein/energy malnutrition, as has been demonstrated in rats [59], the muscle mechanism for maintaining vitamin D status could become less efficient.

## 6. Conclusions

This active cycling of 25(OH)D into and out of skeletal muscle cells explains the long residence time of 25(OH)D in blood. Perhaps then, if ways could be found to optimize this process, the prevalence of some of the diseases linked epidemiologically with low vitamin D status could be minimized. Low circulating levels of vitamins A, E, and K can be related directly to the specific pathology in their function caused by deficiency of these micronutrients. Because vitamin D has been classified as a similar micronutrient, attempts have been made to obtain evidence for a direct link between low concentrations of 25(OH)D in blood and a range of diseases such as diabetes and various cardiovascular and oncology diseases, where there are epidemiological suggestions that low vitamin D status is a causative factor in their etiology. Although the level of 25(OH)D in blood may indicate the adequacy of the vitamin D supply to meet requirements for production of 1,25(OH)_2_D, it is not a direct indicator of the function of that hormone. Because the many regulatory functions of 1,25(OH)_2_D also involve other endocrine and gene expression variables, an epidemiological link between low vitamin D status and a disease process is not necessarily a cause and effect relationship. This is well illustrated by the difficulty of demonstrating health benefits of vitamin D supplementation in many clinical trials [60]. Nevertheless, if ways could be found of optimizing the efficiency of the muscle conservation mechanism for 25(OH)D, perhaps, by a pharmacological agent or some exercise regime, that optimization would ensure that vitamin D status would also be optimized by a process that has evolved to adapt to seasonal changes in the vitamin D supply.

## Figures and Tables

**Table 1:** Comparison of total body 25(OH)D in blood plasma and skeletal muscle.

	Blood Plasma Volume (L)	Muscle Mass (kg)
Total for 70 kg human	2.7–4.3 L [23]	21–26.6 kg ^##^ [22]
25(OH)D concentration	20 µg/L ^###^	1–3 µg/kg ^####^ [21]
Total body 25(OH)D	54–86 µg	21–80 µg

^##^ Assuming body mass of 70 kg, ^###^ Minimum adequate status 25(OH)D concentration, ^####^ Assuming human muscle has similar 25(OH)D concentrations to sheep and cattle.

**Table 2:** Plasma and muscle concentrations of 25(OH)D in outdoor grazing sheep at latitude 33.9° S. Mean values ± Standard Error of the Mean (SEM) [27].

Season	Plasma 25(OH)D_3_	Muscle 25(OH)D
End of summer values (*n* = 5)	10.67 ± 1.65 ng/mL	0.84 ± 0.18 µg/100 g tissue
End of winter values (*n* = 5)	5.36 ± 0.71 ng/mL *	1.82 ± 0.39 µg/100 g tissue *

* Winter values are significantly different from summer values by the *t*-test *p* < 0.05.

**Table 3:** Uptake of [^3^H]-25(OH)D_3_ by differentiated myotubes after 4 h in the presence of varying concentrations of D-binding protein (DBP) (Data reformatted from Reference [28]).

DBP Concentration	0	1 nM	10 nM	100 nM
[^3^H]25(OH)D cpm/mg cell protein	1284 ± 147	1233 ± 67	778 ± 76 *	402 ± 20 *

* Significantly different from 1 nmol/L DBP *p* < 0.001.
